# Using Resident and Faculty Focus Groups to Obtain Stakeholder Input during the ACGME Self-study

**DOI:** 10.1097/pq9.0000000000000186

**Published:** 2019-07-24

**Authors:** Kathryn M. Huggins, Angelina R. Sprewell, Dominique C. Elmore, Meagan W. Shepherd, Tracy L. LeGrow, Marie D. Frazier, Susan L. Flesher

**Affiliations:** From the * Department of Pediatrics, Joan C. Edwards Marshall University School of Medicine, Huntington, W.Va.; † Department of Psychiatry and Behavioral Medicine, Joan C. Edwards Marshall University School of Medicine, Huntington, W.Va.

## Abstract

**Introduction:**

The Accreditation Council for Graduate Medical Education describes steps to guide programs regarding self-study. The process relies heavily on obtaining stakeholder input to validate findings. Focus groups have been noted to provide input and data not elicited using other methods. Our objective was to show the value of focus groups in obtaining a clear picture of stakeholder priorities as they relate to program aims, opportunities, threats, areas for improvement, and sustainability of improvements.

**Methods:**

We conducted a series of focus groups in 2017–2018 at a pediatric residency program with residents and core faculty members. The first session for each group elicited input regarding program aims, opportunities, and threats. The second session focused on evaluating program progress over the past 10 years and identifying strengths and needed areas for improvement. We documented responses obtained during the focus groups and used inductive content analysis methods to identify major themes. We repeated the focus groups every 6 months.

**Results:**

We identified 8 program aims and pinpointed opportunities and threats. Areas for improvement were selected and then presented back to the stakeholders who confirmed their appropriateness. Repetition of the focus groups every 6 months facilitated ongoing measurement of progress and promoted the development of multiple plan, do, study, act cycles for areas of improvement.

**Conclusions:**

Focus groups are an effective way to initiate the self-study process, examine the program aims, opportunities, and threats, and formulate a detailed improvement plan. They are also useful as part of continual evaluation during a dynamic change process.

## Abstract 2

Supplemental Digital Content is available in the text.

## INTRODUCTION

The Accreditation Council for Graduate Medical Education (ACGME) describes 8 steps to guide programs that are organizing their first self-study. These steps include:

(1) assemble the self-study group;
(2) engage program leaders and constituents in a discussion of program aims;
(3) aggregate and analyze data from annual program evaluations (APEs) and the self-study to create a longitudinal assessment of program strengths and areas for improvement;
(4) examine the program’s environment for opportunities and threats;
(5) obtain stakeholder input on strengths, areas for improvement, and threats to prioritize actions;
(6) interpret the data and aggregate the self-study findings;
(7) discuss and validate the findings with stakeholders;
(8) develop a succinct self-study document for use in further program improvement as documentation for the Program’s 10-Year Site Visit.^1^

Although the steps to organizing a self-study are well defined, the process of obtaining stakeholder input, validating findings, and sustaining improvement can vary. Programs often use structured ACGME surveys or questionnaires designed internally as methods to determine the opinions of residents and faculty. Although these do provide valuable information, they require a priori identification of concerns which may not reflect current issues. ACGME allows programs significant freedom in deciding the best way to gain stakeholder input, leading to a current research gap regarding best practices.

**Fig. 1.**
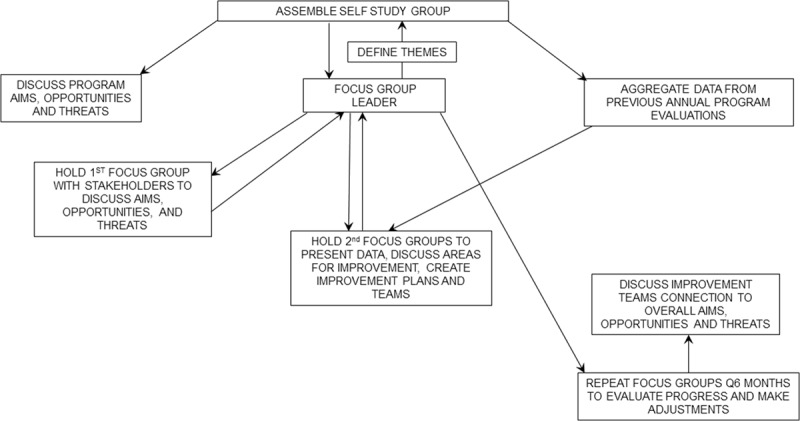
Using focus groups to obtain stakeholder input.

Also, although programs are good at defining goals, they struggle with measuring outcomes and maintaining improvement. While setting goals is fairly easy, it is much more difficult to finish them.^2^ Our goal is to describe how we used focus groups to gather stakeholder input and how the groups helped assure we defined measurable outcomes and sustained our improvements (Fig. 1).

Our hypothesis was twofold. We believed that focus groups would be as follows:

(1) An excellent model for obtaining stakeholder input regarding program aims, opportunities, threats, and areas for improvement. (We based this on the fact that the utilization of focus groups has long been known to aid in gaining a deeper understanding of a particular problem, program, or phenomenon.^3,4^)
(2) An effective approach for defining and sustaining measurable outcomes.

## METHODS

The study was conducted in 2017–2018 at a pediatric residency program within an academic medical center. IRB approval was not required as this project was not Human Subjects Research.

We conducted a series of focus groups with residents and faculty members serving as focus group participants. All 18 categorical and 8 medicine/pediatric residents enrolled in the program and 20 core faculty members, as defined by ACGME standards,^5^ were invited by email to participate in the focus groups and all elected to do so. We conducted focus groups for residents and faculty members separately, and each group was seen twice within 1 week at the beginning of the self-study and then again for follow-up at 6 and 12 months after the initial focus group sessions. All focus groups were 1 hour in length and were facilitated by a doctoral-level psychologist who had experience with focus groups. At session one and session two, a psychology graduate student was also present to help with data collection. At each session, participants received feedback regarding prior sessions.

### Session One

The first session for each group consisted of a series of questions designed to elicit responses regarding program aims, opportunities, and threats. At the beginning of this session, the program director provided a presentation explaining the ACGME self-study process with the specific directions provided on the ACGME website for defining aims, opportunities, and threats. We explained that aims are a long-term strategic view of key expectations for the program that show what kind of physicians the program aims to produce.^1^ To increase the transparency of stakeholder responses, the program director was not in attendance after the presentation.

### Session Two

During the second session, each group reviewed and evaluated the past 10 years of program data, focusing on program growth and responses to areas of need. Before the second session, participants had received copies of 10 years of APEs in addition to an aggregate summary of all 10 years to facilitate discussion and to provide a historical perspective for both residents and junior faculty.

To facilitate thinking more broadly about the proposed areas for improvement, we asked both resident and faculty groups to identify a “wish list” of things that would make the program better. In addition to questions regarding the growth and development of the residency program, the facilitator asked faculty about their perceptions regarding faculty-specific needs, including faculty development options.

We recorded responses obtained during the focus groups in writing, and we used inductive content analysis methods to identify major themes across all questions. Inductive content analysis is a qualitative method used to identify themes and develop hypotheses/theory by repeated examination of raw data. These data can include written documents, interviews, etc. Using this method, we make no a priori hypotheses, and themes are seen as emerging from data in such a way that it tells the story of what is being studied. Data were coded based on emerging themes, and a validity check was done by having individuals not involved in the focus groups (psychology graduate students) sort the data into the identified themes/categories. Also, themes identified in the focus groups were shared with the residents and core faculty to provide closed-loop communication and to provide an additional validity check with the focus group analysis in terms of identified program aims, opportunities, threats, strengths, and areas for improvement. The focus group leader provided a written report of each session to the self-study group.

Once we established areas for improvement, we developed improvement teams. Each team was led by at least one resident and faculty volunteer. Because other programs have had difficulty with measuring outcomes and maintaining improvement, team leaders were asked to oversee these issues through individual quality improvement initiatives.

### Biannual Follow-up Sessions

We conducted additional focus group sessions at 6-month intervals. The focus during these sessions was on reviewing the identified areas of improvement and identifying successes and challenges of the improvement teams and needed adjustments to the improvement plans. At each of these follow-up sessions, participants were encouraged to suggest additional measurable outcomes.

Following the first follow-up session 6 months into the process, participants reported primarily positive progress and had little to say regarding challenges. At the follow-up session at 12 months, the program director made a short presentation at the beginning of both the resident and faculty groups to encourage more feedback regarding challenges and ways to measure progress.

## RESULTS

The focus groups identified 8 program aims:

- Provide varied learning opportunities to train individuals to be outstanding general pediatricians and to prepare those who choose fellowship training appropriately.
- Plan and execute educational experiences that result in the mastery of pediatric milestones and core competencies and success on the pediatric board exam.
- Mold a commitment to self-directed learning and participation in research.
- Serve the needs of underserved individuals in this part of the Appalachian region both directly and by fostering an understanding of rural populations and of the challenges of providing healthcare in small communities.
- Serve the needs of our infants with neonatal abstinence syndrome both directly and by teaching about the comprehensive needs of this population.
- Provide a “family-like” atmosphere that is positive and supportive, where physician wellness and self-care are important.
- Prepare residents to advocate for children, including involvement in legislative advocacy and formation of health policies.
- Teach residents to understand the business of medicine.

As per ACGME self-study instructions,^1^ these aims were then approved by the self-study group that included the department chair, the program director, and the associate program director. These aims were also approved at the institutional level by the graduate medical education office designated institutional officer before being submitted to ACGME. Also, the self-study group identified opportunities and threats and selected 5 areas for improvement based on themes from both the resident and core faculty focus group. We included most areas for improvement that emerged in the focus group report. The only ones that were not included were those that the self-study group determined would not be best addressed by an improvement team. For example, one area involved concern about a specific faculty member which the group determined would be appropriately addressed directly by the department chair. Due to input from the faculty focus group, we also included faculty development in the improvement plan. Finally, the self-study group added 2 more areas based on the more global program aims, as the areas identified by the focus groups tended to be quite detailed and specific. This addition resulted in a total of 8 improvement teams, 6 directly identified based on areas for improvement which were defined in Session Two, including one to improve faculty development, and two based directly on program aims from Session One. Supplemental Digital Content, Table 1, available at http://links.lww.com/PQ9/A119, lists the 8 improvement teams and details baseline data, action plans, 6- and 12-month outcomes, and next steps. Supplemental Digital Content 2, Table 2, available at Supplemental Digital Content 1, http://links.lww.com/PQ9/A106. includes additional data regarding the faculty development improvement team.

Each improvement team was assigned resident and faculty leaders who tracked and measured outcomes and reported back to the self-study group. The first 6-month follow-up showed many positive outcomes. After encouragement to do so, the second 6-month session also included more input on what was not going well and could be further improved and new ideas for measurement. This expanded input allowed for the creation of additional plan, do, study, act cycles by the improvement teams.

## DISCUSSION

The definition of program aims and the elucidation of specific opportunities and threats is a natural fit for focus group methodology as it allows for an active process that can capture the nuances of a program. Although the use of questionnaires is helpful and is one of the many elements we use in our APEs, it requires to a greater extent, a priori identification of specific areas of inquiry that may or may not reflect current program norms. This factor is especially important as the ACGME encourages programs to develop their own identities. A secondary gain in the utilization of focus groups is that participants are truly “heard,” and this can allow for greater stakeholder participation and buy-in than is typical when less dynamic methods are used.^6,7^

Focus groups are seen as allowing for greater flexibility and the ability to gather a richer set of data due to participant interactions with each other during the focus group process.^8^ Basch^9^ suggests that the data that are collected could be considered similar to formative evaluations in that they do not rate or score a program but rather identify the current state of the program and offer guidance in terms of opportunities for growth and change. Participants in this project received feedback from the previous sessions and were able to utilize that feedback as they went into the remaining sessions. This feedback allowed for the process to remain focused and to dig deeper into the areas identified as important.

For example, our second area for improvement involved the hiring of a faculty person triple-boarded in pediatrics, psychiatry, and child psychiatry. Part of the goal was that during the behavior development rotation, the pediatric resident would spend 2 mornings a week with this triple-boarded faculty member in her child psychiatry clinic. At the 12-month follow-up focus group, we realized residents were being assigned to work with this faculty member during her time seeing routine general pediatric patients for nonpsychiatric visits instead of working with her in her psychiatry clinic. Perhaps, one of the most important things the ongoing focus groups accomplished was revealing what was not working and keeping us accountable to the improvement process.

Another important success was in encouraging participants to continue to develop new outcome measures. For this same improvement plan, the 12-month follow-up focus group recommended including in-training exam scoring in this specific area of mental healthcare.

The major limitation of this study is that we did not have a comparison group using other methods besides focus groups. We do have some feeling for comparison as the self-study is a natural expansion of the APE. Every year, we consider data including faculty and resident ACGME surveys, internal in-house faculty and resident electronic surveys, resident progression on milestones, graduate surveys, in-training exam scores, American Board of Pediatrics first-time pass rates, and confidential resident evaluations of faculty and rotations. The resident members also communicate directly with all residents and supply further information by word of mouth. In the setting of our experience with multiple methods, we anecdotally found that focus groups were a unique approach to identifying more varied and detailed areas for improvement.

## CONCLUSIONS

Focus groups are an effective way to gather stakeholder input during the self-study process. They allow a program to examine aims, opportunities, threats, and strengths, and to formulate a detailed improvement plan. When guided to do so, they can establish measurable outcomes, and when conducted in an ongoing fashion, they facilitate staying on track during a dynamic improvement process.

## DISCLOSURE

The authors have no financial interest to declare in relation to the content of this article.

## Supplementary Material
