# Supplementary material for: Using Resident and Faculty Focus Groups to Obtain Stakeholder Input during the ACGME Self-study
Source: Pediatr Qual Saf. 2019 Jul 24;4(4):e186. doi: 10.1097/pq9.0000000000000186 (PMC6708642; doi:10.1097/pq9.0000000000000186)
Supplement: Supplementary file 1 [file pqs-4-e186-s001.docx]

| **Area for improvement** | **Baseline data/Initial**  **focus groups** | **Action plan/PDSA cycles** | **6-month outcomes** | **12-month outcomes** | **Future actions/PDSA cycles** |
| --- | --- | --- | --- | --- | --- |
| Improve resident attendance at deliveries during NICU rotation | Residents were attending 29% of NICU response high risk deliveries occurring during resident NICU working hours | Placed sign by elevator to delivery suite saying, ‘Don’t forget the resident!’  Residents recorded they were there to assure accuracy of count  Added one night per month to NICU resident schedule/previously all days  Placed button by elevator that alerts a device on resident lanyard  Enlisted help of unit clerk to call resident  Initiated nurse reward system of pizza parties for attendance over 70% | 74%  Increase significant p<0.001 | 79% | Continue nurse reward system |
| Increase resident and faculty comfort and experience with managing basic mental health conditions such as depression and anxiety | Residents and faculty reported reluctance on the part of some general pediatric attendings to treat common pediatric psychiatric conditions resulting in loss in training for residents in the clinical setting | Hired a peds/psych/child psych triple board faculty person  Increased resident didactic time for behavioral and mental health  Increased emphasis on availability of child and adolescent psychiatry as part of resident individualized curriculum  Scheduled residents time with triple board faculty in her psychiatry clinic  Started quarterly question and answer session with the triple board faculty during protected resident lecture block time  Triple board faculty provided grand rounds on depression | Residents reported increased comfort at 6-month focus group | Residents reported increased comfort at 12-month focus group  On the in-training examination our residents are now above national average in the mental health and behavioral section at every training year level:  Ours National  Year 1-88 87  Year 2-96 91  Year 3-93 92  (Comparable baseline data not available) | Have triple board faculty increase focus on a specific topic during quarterly question and answer session as residents reported there were not many questions asked. |
| Increase resident exposure to different attending practices in resident clinic | Residents and faculty noted that it would be nice to rotate the residents’ continuity clinics each year to allow them exposure to different attendings because each attending has strengths and weaknesses | Rotated resident continuity clinic each year of residency  Added nursery attendings to clinic in afternoons, widening exposure to different attendings | Residents who experienced schedule change were surveyed:  47% experience definitely improved  52% experience somewhat improved  64% said rotating system should be continued | Residents and faculty at 12-month focus group continued to report this as positive change | Have clinic residents rotate through NowCare (attending run walk-in clinic) in the evenings |
| Increase new intern comfort with providing anticipatory guidance in resident clinic | Residents and faculty noted outpatient clinic is difficult for new interns as they are not sure what anticipatory guidance to provide patients  Second year class was surveyed and asked to answer questions based on the first 4 months of their intern year:  78% noted less than 25% of time anticipatory guidance handout reviewed with attending prior to patient visit.  22% noted this occurred 25-50% of the time  In response to how confident they felt providing anticipatory guidance:  0% strongly confident  11% somewhat confident  11% neutral  67% somewhat not confident  11% not at all confident | Provided individual Bright Futures Flip Chart to new interns  Provided copy of Med Study books to clinic for interns to read anticipatory guidance  Senior resident met with interns during orientation and discussed how to give anticipatory guidance  Clinic attendings were instructed to review anticipatory guidance handouts with the intern prior to the intern going into patient room. Also, attendings instructed to go into room initially with interns rather than just coming in at the end of the patient visit. | Interns surveyed:  38% noted less than 25% of time anticipatory guidance handout reviewed with attending prior to patient visit.  38% noted this occurred 25-50% of the time.  25% noted this occurred 75-100% of time  In response to how confident they felt providing anticipatory guidance:  12.5% strongly confident  62.5% somewhat confident  12.5% neutral  12.5% somewhat not confident  0% not at all confident  Increase in confidence significant (p=.002) | Residents reported increased comfort at 12-month focus group  On the in-training examination our residents are now close to or above national average in the preventive pediatrics/well care section at every training year level:  Ours National  Year 1-72 68  Year 2-73 75  Year 3-77 78  (Comparable baseline data not available)  This in-training exam data regarding preventive pediatrics/well care was further broken down to reflect specific data about three questions on anticipatory guidance as follows. Percent of our residents answering correctly  Question 1: 100%  Question 2: 89%  Question 3: 89% | New issue identified at 12-month focus group while discussing anticipatory guidance. While residents are comfortable with anticipatory guidance, they expressed lack of comfort with billing in the clinic. This is a new area for improvement. |
| Provide guidance to rising second years regarding expectations as seniors | Residents and faculty noted the transition to be a senior resident was often difficult as residents didn’t understand expectations  Third year residents were surveyed regarding how well prepared they felt prior to becoming a senior resident. 33% responded - not prepared at all. 67% responded – somewhat prepared.  100% responded that a meeting with a senior resident to discuss expectations would have been helpful. | Pediatric hospitalist, neonatologist and selected senior resident met to discuss goals and expectations they had of rising senior residents  These expectations were passed on by the senior resident to rising senior residents at a meeting with a question and answer session | Residents who attended the meeting to discuss expectations were surveyed prior to and after the meeting regarding how well prepared they felt.  Prior to meeting:  50% - not prepared at all  25% - somewhat prepared  25% - well prepared  After meeting:  25% - somewhat prepared  75% - well prepared  100% felt the discussion was helpful | Residents and faculty at 12-month focus group continued to report this as positive intervention | Continue yearly  Continue to survey residents before and after sessions to gather more data to achieve significance |
| Serve the needs of underserved individuals in this part of Appalachia both directly and by fostering an understanding of rural populations and challenges of providing health care in small communities | Focus groups identified this as a global program aim. The self-study group added as area for improvement as they felt we could do a better job of understanding and preparing for challenges.  Senior resident on improvement team called seven former graduates who are practicing in rural areas and questioned them regarding their feeling of preparedness for their practice. All relayed for most part they felt well prepared. Four said more presence at deliveries would be helpful. Two said they wished they had more experience managing complex, chronic illnesses. | Held two grand rounds followed by question and answers lunches with 4 total previous graduates who are practicing in rural areas  Resident schedule adjusted to assure all residents have equal opportunity to be on the clinical experience with Children’s Health Fund rural van which travels to schools in rural areas.  Started project to increase resident exposure to normal low risk deliveries during newborn nursery rotation with the goal of residents attending at least 10 deliveries. (This is a separate project from the prior mentioned increasing attendance at high risk deliveries during NICU rotation) At this point have:  -obtained Spectralink phone for nursery resident  -permission obtained from obstetricians  -spoke to residents about importance and encouraged them to go to labor and delivery daily and ask to be called  -sign posted to remind labor and delivery patient care assistant to call the pediatric resident  -resident to go the labor and delivery during their free afternoons and talk to OB residents about being called or staying around for deliveries  Added high fidelity infant simulator to block didactic schedule. | Residents and faculty at 6-month focus group relayed grand rounds extremely helpful and they wish to continue  These areas identified by previous graduates at 1st grand rounds:  Need to feel competent with neonatal resuscitation  Need for more mock codes  Need for residents to focus on management of common things – (constipation, reflux, seizures, murmurs), when they are learning from subspecialists | Residents and faculty at 12-month focus group relayed grand rounds extremely helpful and they wish to continue  These areas identified by previous graduates at 2^nd^ grand rounds:  Emphasized they did get good education and saw large volume and wide amount of pathology  Encouraged residents to stand up for what they know is correct management, not feel they must do what other practitioners in their communities are doing.  Recommended residents take every opportunity to see one more patient, learn one more thing  Said if they could do it over, they would spend more time in ER, more time responding to level one traumas, codes, etc.  Said they would spend more time with subspecialists | Continue rural grand rounds yearly  Monitor outcomes of delivery attendance during NBN rotation  Add more high-fidelity infant and child simulator mock codes to block didactic schedule |
| Serve the needs of our infants with neonatal abstinence syndrome both directly and by teaching about the comprehensive needs of this population. | Focus groups identified this as a global program aim. The self-study group added as area for improvement since although residents had clinical experience with this, the didactic education was lacking | Grand rounds on neonatal abstinence syndrome held  Incorporated education on neonatal abstinence syndrome into fetus and newborn infant didactic block  Changed attending scheduling in unit caring for neonatal abstinence syndrome to provide more consistency and specialization |  | Residents at 12-month follow up expressed concerns about not knowing treatment protocols and noted protocols between NICU and unit caring for neonatal abstinence syndrome babies are not consistent | Repeat grand rounds on treatment protocols.  Provide resident handouts on treatment protocols |
| All faculty members involved in education of residents should participate in programs to enhance the effectiveness of their skills as educators | Faculty expressed they are more interested in learning clinical updates than faculty development topics which they consider to be less relevant. They also noted faculty development needs to be pediatric specific, practical and useful, and that grand rounds is a good time to for them to be able to attend | We created a novel approach that integrates faculty development with education regarding clinical medicine topics  Provided grand rounds using this approach which discussed current expert opinion regarding the management of septic arthritis and osteomyelitis in children. We then discussed an actual recent osteomyelitis case where several areas of contention arose during management. At this point we discussed conflict resolution tying this back into the case discussed | Assessed response using a questionnaire and majority of attendees found the integrated format helpful. See Table 2 for specific data | Faculty at 12-month follow up focus group continued to express they were excited about this approach finding it very practical | Repeat novel approach during additional grand rounds and obtain more questionnaire data |

**Table 1 Improvement Team Data**
