# Supplementary material for: Using Resident and Faculty Focus Groups to Obtain Stakeholder Input during the ACGME Self-study
Source: Pediatr Qual Saf. 2019 Jul 24;4(4):e186. doi: 10.1097/pq9.0000000000000186 (PMC6708642; doi:10.1097/pq9.0000000000000186)
Supplement: Supplementary file 2 [file pqs-4-e186-s002.docx]

| **Post-Exercise Assessment Questions** | **Medical Students** | **Residents** | **Faculty** |
| --- | --- | --- | --- |
| Q1. I found this integrated format, which included both clinical content and faculty development, to be helpful in regard to clinical content…. | 7 | 13 | 6 |
| Q2. I found this integrated format, which included both clinical content and faculty development, to be helpful in regard to faculty development…. | 8 | 12 | 6 |
| Q3.* My interest in attending this lecture was increased upon the knowledge that it included both clinical content and faculty development. | 2 | 10 | 5 |
| Q4. In the future, I plan to utilize skills (both clinical and professional) that I have learned from this lecture. | 9 | 15 | 5 |
| Q5. In the future, I would like to attend more lectures that have integrated both clinical content and faculty development. | 8 | 14 | 6 |
| **Total Responders** | 9 | 15 | 7 |

**Table 2: Data from Novel Approach of Integrated Faculty Development**

Table 2 Explanation. Lecture attendees were asked to complete 5 questions reflecting their attitudes toward the exercise. Answers were assessed with a 5-point Likert scale, with 1 equivalent to Strongly Disagree, 2 equivalent to Disagree, 3 equivalent to Neither Agree nor Disagree, 4 equivalent to Agree, and 5 equivalent to Strongly Agree. The data was then dichotomized and the above numbers reflect those attendees who chose either Agree or Strongly Agree.

*For question 3, six medical students, five residents, and one faculty member chose Neither Agree nor Disagree.
